# Cooperation between HMGA1, PDX-1, and MafA is Essential for Glucose-Induced Insulin Transcription in Pancreatic Beta Cells

**DOI:** 10.3389/fendo.2014.00237

**Published:** 2015-01-13

**Authors:** Biagio Arcidiacono, Stefania Iiritano, Eusebio Chiefari, Francesco S. Brunetti, Guoqiang Gu, Daniela Patrizia Foti, Antonio Brunetti

**Affiliations:** ^1^Department of Health Sciences, University “Magna Græcia” of Catanzaro, Catanzaro, Italy; ^2^Department of Medical and Surgical Sciences, University “Magna Græcia” of Catanzaro, Catanzaro, Italy; ^3^Department of Cell and Developmental Biology, Center of Stem Cell Biology, Vanderbilt Medical Center, Nashville, TN, USA

**Keywords:** HMGA1, PDX-1, MafA, insulin gene, beta-cells, transcription, diabetes

## Abstract

The high-mobility group AT-hook 1 (HMGA1) protein is a nuclear architectural factor that can organize chromatin structures. It regulates gene expression by controlling the formation of stereospecific multiprotein complexes called “enhanceosomes” on the AT-rich regions of target gene promoters. Previously, we reported that defects in HMGA1 caused decreased insulin receptor expression and increased susceptibility to type 2 diabetes mellitus in humans and mice. Interestingly, mice with disrupted *HMGA1* gene had significantly smaller islets and decreased insulin content in their pancreata, suggesting that HMGA1 may have a direct role in insulin transcription and secretion. Herein, we investigate the regulatory roles of HMGA1 in insulin transcription. We provide evidence that HMGA1 physically interacts with PDX-1 and MafA, two critical transcription factors for insulin gene expression and beta-cell function, both *in vitro* and *in vivo*. We then show that the overexpression of HMGA1 significantly improves the transactivating activity of PDX-1 and MafA on human and mouse insulin promoters, while HMGA1 knockdown considerably decreased this transactivating activity. Lastly, we demonstrate that high glucose stimulus significantly increases the binding of HMGA1 to the insulin (*INS*) gene promoter, suggesting that HMGA1 may act as a glucose-sensitive element controlling the transcription of the *INS* gene. Together, our findings provide evidence that HMGA1, by regulating PDX-1- and MafA-induced transactivation of the *INS* gene promoter, plays a critical role in pancreatic beta-cell function and insulin production.

## Introduction

Production and secretion of insulin from beta cells of the pancreas maintain glucose homeostasis in vertebrates (from fish to human). Absence or dysfunction of beta cells results in diabetes mellitus, a set of genetically heterogeneous disorders featured by high blood sugar and related complications. The majority of diabetes is type 2 diabetes, which is featured by defects in both pancreatic insulin secretion (beta-cell dysfunction) and peripheral insulin action (insulin resistance) (1, 2). Therefore, understanding beta-cell generation and insulin production/secretion holds keys to understand and potentially cure diabetes.

Insulin production in beta cells is controlled at both transcriptional and translational levels (3). High glucose upregulates both insulin transcription and preproinsulin translation. To this end, glucose regulates proinsulin translation by altering the phosphorylation status of eukaryotic initiation factor 2α (4), while it promotes insulin transcription by activation of several beta-cell specific transcription factors (5). Yet, direct connections between high glucose and activation of beta-cell transcription factors have not been established.

Regulation of insulin (*INS*) gene expression has been characterized by several groups. A 500-bp stretch of DNA upstream of the *INS* transcription start site controls its transcription. Several *cis*-acting regulatory sequences reside in this region, with highly conserved A3, C1, E1, and CRE *cis*-acting regulatory elements (6, 7). These promoter regions bind ubiquitous factors and a pool of beta-cell specific transcription factors (8–14). Among these, the pancreatic and duodenal homeobox factor-1 (PDX-1) and MafA (V-maf musculoaponeurotic fibrosarcoma oncogene homolog A) are the most notable factors. PDX-1 forms multiprotein complexes with several beta-cell transcription factors to synergistically activate the *INS* promoter (7). It also mediates a role of high glucose in the up-regulation of *INS* transcription in humans and rodents (15–22). Consequently, targeted disruption of *PDX-1* gene in beta cells leads to beta-cell dysfunction and overt diabetes in mice (23), whereas mutations in *PDX-1* have been linked to pancreatic agenesis and diabetes in humans (24, 25). Similarly, MafA activates *INS* transcription through the E1 and C1 elements of the *INS* gene promoter (26–31). Its inactivation results in immature beta cells with lowered insulin expression and secretion and severe glucose intolerance (32, 33).

High-mobility group AT-hook 1 (HMGA1) is an architectural transcription factor that binds to the minor groove of AT-rich regions of DNA (34). This binding alters the DNA conformation and recruits transcription factors to the transcription initiation site to assemble stereospecific DNA–protein complexes (so-called enhanceosomes) that drive gene transcription (35, 36). The fact that HMGA1 is expressed at high levels in most cell types during embryonic development suggests its wide-spread roles in gene expression and function (37). We have previously demonstrated that loss of function in HMGA1 in both human and mice compromised pancreatic endocrine function and resulted in diabetes (38). We further showed that binding of PDX-1 to the *INS* gene promoter was reduced in nuclear extracts from *HMGA1*-deficient mice, in which PDX-1 protein expression was unaffected compared with wild-type animals (38). Furthermore, perturbation of HMGA1 in the insulin-secreting beta-cell line INS-1 transfected with an antisense expression vector specific for *HMGA1* impaired glucose-stimulated insulin secretion, suggesting that HMGA1 may have a direct role in insulin production and pancreatic islet development through PDX-1 and/or other nuclear molecular partners. In this context, it is noteworthy that *Ins* gene transcription is reduced in *Hmga1*-deficient mice, in which pancreatic islets are up to 80% smaller compared to those of wild-type mice (38), and reports of others have suggested that HMGA1 binds to the A3/4 region of the *INS* gene promoter (14).

In this study, we investigated whether HMGA1 can directly activate the *INS* gene by synergizing with PDX-1 and MafA. Additionally, the effect of glucose on HMGA1 affinity to the *INS* gene promoter was also investigated.

## Materials and Methods

### Glutathione S-transferase pull-down assay and co-immunoprecipitation

Human ^35^S-labeled PDX-1 and MafA proteins were synthesized *in vitro* using the TNT-T7 quick-coupled transcription/translation system (Promega), as previously reported (36). Glutathione S-transferase (GST)-tagged human HMGA1 was obtained using the pcDNA1-GST/HMGA1 expression vector, a kind gift from D. Thanos (Biomedical Research Foundation, Academy of Athens, Athens, Greece). For direct coupling of antibody to protein A-Sepharose beads (GE Healthcare), an anti-HMGA1 polyclonal antibody was mixed with beads and bound for 1 h with rotation at room temperature, as described previously (36). Antibody-coupled protein A beads were washed twice in phosphate-buffered saline and used in immunoprecipitation studies. Briefly, aliquots of INS-1 cell nuclear extract, HMGA1, PDX-1, or MafA together were incubated for 3 h with rotation at 4°C with 10 μl of antibody-coupled protein A beads. Beads were recovered by gentle centrifugation and washed three times with 500 μl of NETN wash buffer [0.1% NP-40, 150 mM NaCl, 1 mM EDTA, 50 mM Tris-HCl (pH 8.0)]. Protein was removed from the beads by boiling in sample buffer for 5 min and analyzed by SDS-PAGE and immunoblotting (39). Antibodies used for these studies were as follows: anti-HMGA1 (39), anti-PDX-1 (40), and anti-MafA (Abcam).

### Cell cultures and nuclear extracts

Human embryonic kidney (HEK) 293 cells and human epithelial carcinoma (HeLa) cells were cultured in DMEM (Invitrogen) supplemented with 10% fetal bovine serum (FBS), 2 mM glutamine, penicillin (100 U/ml), and streptomycin (100 μg/ml) in a humidified 5% CO_2_ atmosphere at 37°C. INS-1 rat insulinoma cells were cultured in RPMI-1640 medium supplemented with 10% FBS, 2 mM glutamine, penicillin (100 U/ml), streptomycin (100 μg/ml), 50 μM beta-mercaptoethanol, and 100 mM HEPES buffer (Sigma). Nuclear extracts were prepared from cultured cells as described previously (41, 42). For each extract, an equal number of nuclei were homogenized, and the final protein concentration in the extracts was determined using the colorimetric assay of Bradford (Bio-Rad).

### Plasmid vectors, small interfering RNA, and transient transfection

The promoter regions of human and mouse *INS* genes were amplified from genomic DNA, using modified specific primers (Table 1). Sequence-verified promoters were then cloned into the pGL3-basic reporter vector (Promega) to generate the human (*phINS-Luc*) and mouse (*pmINSII-Luc*) luciferase (*Luc*) reporter plasmids. The expression plasmids were as follows: pcDNA3/HA-HMGA1 (a generous gift from G. Manfioletti, University of Trieste, Italy); pcDNA3-MafA (a generous gift from P. Gold, Gold Biotechnology, Inc., St. Louis, MO, USA); pSG5-ATG-hPDX-1 (a generous gift from C. V. Wright, Vanderbilt University Medical Center, Nashville, TN, USA). For gene silencing experiments, mouse and human anti-HMGA1 small interfering RNAs (siRNAs) (Santa Cruz Biotech) were used. In all knockdown experiments, cells were transfected with 100 pmol of HMGA1 siRNA in 6 well plates, and incubated without further treatment for 72 h before being used in subsequent analyses. Transient transfections of cultured cells were carried out using the Lipofectamine 2000 method (Invitrogen), and *Luc* activity was assayed 48–96 h later, using the dual-luciferase reporter assay system (Promega) (43).

**Table 1:** **Primers used for plasmid construction of human and mouse *INS* promoter-containing vectors**.

Name	Sequence (5′–3′)	Position^a^	Restriction site
Human *INS* for	accaccttggtacctccatggcggcatctt	−810/−781	*Kpn*I
Human *INS* rev	tgtgtagaagaagcttcgttccccg	+366/+390	*Hin*dIII
Mouse *InsII* for	agaaaggtttggtacctggaatagagc	−683/−653	*Kpn*I
Mouse *InsII* rev	gtagaagaagcttcgctccccaca	+301/+324	*Hin*dIII

*^a^From the transcription start site. Modified bases are underlined*.

### Glucose stimulation of insulin release

INS-1 cells (ATCC) were cultured in RPMI-1640 medium (GIBCO) supplemented with 10% FBS, 2 mM glutamine, penicillin (100 U/ml), streptomycin (100 μg/ml), 50 μM beta-mercaptoethanol, 100 mM HEPES, in a humidified 5% CO_2_ atmosphere at 37°C. For insulin secretion, INS-1 cells were transiently transfected at 60% confluence with HMGA1 siRNA or negative control siRNA as above. Seventy-two hours later the tissue culture medium was switched to fresh medium containing 5 mmol/l glucose and the cells were grown for additional 18 h. After this, the cells were washed with Hanks’ balanced salt solution (HBSS) containing 140 mM NaCl, 4.7 mM KCl, 1.2 mM KH_2_PO_4_, 1.16 mM MgSO_4_, 20 mM HEPES, 2.5 mM CaCl_2_, 25.5 mM NaHCO_3_, 0.2% BSA, and further incubated for 2 h in either low (3 mM) or high (15 mM) glucose condition. Then, the insulin concentration in the medium was measured by a commercial rat insulin ELISA kit (Mercodia Inc.).

### Chromatin immunoprecipitation

Chromatin immunoprecipitation (ChIP) was performed in INS-1 cells as described previously (38). As soon as the cells reached confluence, they were incubated in HBSS buffer with 3 or 15 mM glucose for 30 min at 37°C. Then, the cells were washed with 1× PBS and fresh RPMI was added. DNA–protein complexes were cross-linked by adding formaldehyde diluted to 1% final concentration for 10 min at room temperature, followed by blocking with glycine for 2 min. Cells were washed twice with cold PBS and lysed on ice using SDS lysis buffer (1% SDS, 10 mM EDTA, 50 mM Tris pH 8). Chromatin samples were sonicated on ice and the formaldehyde-fixed DNA–protein complexes were immunoprecipitated with anti-HMGA1 antibody (Santa Cruz Biotech) and the following sequence-specific primers for the rat *InsI* gene promoter were used for PCR amplification of immunoprecipitated DNA, using PCR ready-to-go beads (GE Healthcare). Rat *InsI* (NC_005100.3): for 5′-CTGGGAAATGAGGTGGAAAA-3′ (−328/−308 from the trasncription start site); rev 5′-AGGAGGGGTAGGTAGGCAGA-3′ (−108/−88 from the trasncription start site). PCR products were electrophoretically resolved on 1.5% agarose gel and stained with ethidium bromide staining solution. PCR products were confirmed by sequence analysis.

### Real-time PCR

For qRT-PCR, total cellular RNA was extracted from INS-1 cells using the RNAqueous-4PCR kit and subjected to DNase treatment (Ambion). RNA levels were normalized against *18S* ribosomal RNA in each sample, and cDNAs were synthesized from 2 μg of total RNA using the RETROscript first strand synthesis kit (Ambion). Primers for rat *InsI*, *HMGA1*, *PDX-1*, *MafA*, and *Rps9* were designed according to sequences from the GenBank database (Table 2). A real-time thermocycler (Eppendorf Mastercycler ep realplex ES) was used to perform quantitative PCR. SYBR Green fluorescence was measured, and relative quantification was made against the *Rps9* cDNA used as an internal standard (44). All PCR reactions were done in triplicate.

**Table 2:** **Gene-specific primers for Real-Time PCR**.

Name	Sequence (5′–3′)	Size (bp)	Accession no.
Rat *RPS9* for	tttgtcgcaaaacctatgtgacc	175	NM_031108.3
Rat *RPS9* rev	ttctcgtccagcgtcaacag		
Rat *HMGA1* for	aaagttaccacaactccggg	173	XM_006256160.1
Rat *HMGA1* rev	agcagggcttccagtcccag		
Rat *PDX-1* for	aaatccaccaaagctcacgc	188	NM_022852.3
Rat *PDX-1* rev	aagttgagcatcactgccagc		
Rat *MafA* for	aggaggaggtcatccgactg	113	XM_006241903.1
Rat *MafA* rev	cttctcgctctccagaatgtg		
Rat *InsI* for	gacccgcaagtgccacaa	101	NM_019129
Rat *InsI* rev	tccacaagccacgcttctg		

### Statistical analysis

All calculations were performed with SPSS 20.0 statistical software (SPSS Inc.). Mean values were compared with *t*-tests. A *p*-value <0.05 (two-tailed) was considered significant.

## Results

### HMGA1 physically interacts with PDX-1 and MafA *in vitro* and *in vivo*

**Figure 1:**
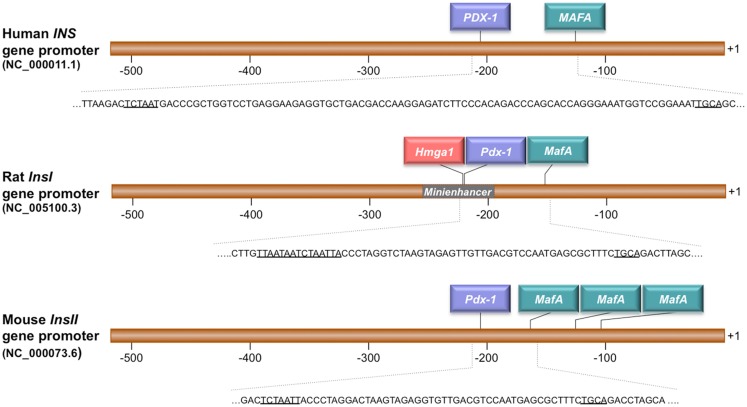
**Schematic representation of the human (*INS*), rat (*InsI*), and mouse (*InsII*) promoter regions**. HMGA1, PDX-1, and MafA binding sites are underlined in each gene sequence. The numbers indicate positions in base pairs relative to the transcriptional start site (+1). The rat insulin mini-enhancer element E2A3/4 is indicated.

Figure 1 shows a schematic representation of the promoter region of human, mouse, and rat *INS* genes. Binding sites for HMGA1, PDX-1, and MafA were identified within the promoter of the rat *Ins* gene by sequence analysis with MatINspector (version 8.1, Genomatix, http://www.genomatix.de/). Bioinformatics analysis of the human and mouse *INS* promoters predicted no HMGA1 binding sites, although the existence of non-recognizable DNA motifs cannot be completely excluded. Instead, binding sites for PDX-1 and MafA were predicted in these promoters as for the rat *Ins* gene. Consistent with this DNA promoter analysis, the existence of HMGA1 in a cocomplex with PDX-1 was demonstrated previously by us and others in supershift experiments in electrophoretic mobility shift assays using anti-HMGA1 and anti-PDX-1 antibodies, in the presence of the rat *InsI E2A3/4* mini-enhancer DNA (14, 38). In both investigations, binding of PDX-1 to this promoter element was increased in the presence of HMGA1, suggesting a functional cooperation between HMGA1 and PDX-1 on the *INS* promoter.

**Figure 2:**
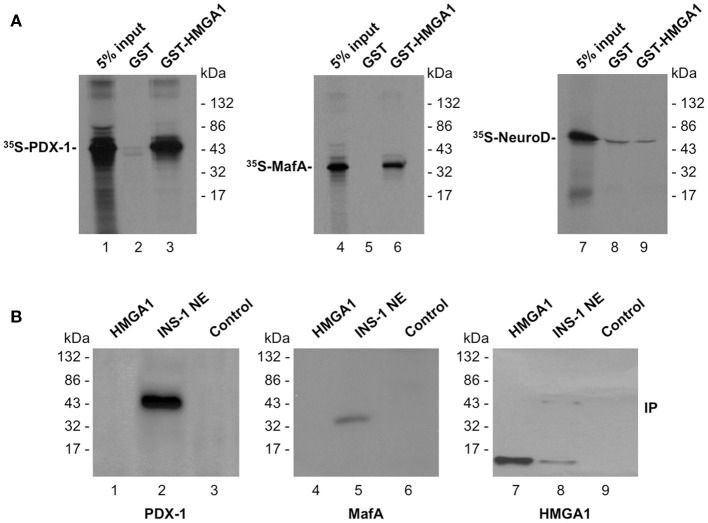
**Physical association between HMGA1, PDX-1, and MafA**. **(A)** SDS-PAGE of ^35^S-PDX-1, ^35^S-MafA, and ^35^S-NeuroD bound to GST or GST-HMGA1 resin (lanes 1–9). In lanes 1, 4, and 7 labeled protein was added directly onto the gel without binding to and elution from GST protein resin. **(B)** Immunoprecipitation (IP) of HMGA1, PDX-1, and MafA by using the anti-HMGA1 antibody followed by immunoblotting with the anti-PDX-1 antibody (lanes 1–3), the anti-MafA antibody (lanes 4–6), or the anti-HMGA1 antibody (lanes 7–9) after reprobing the same transfer. Lanes: 1, 4, and 7, 10 ng of pure HMGA1, 2, 5, and 8, INS-1 nuclear extract (NE; 500 μg). In lanes 1, 4, and 7, protein was directly applied to the gel without binding to and elution from protein A beads. To prove specificity, pure Sp1 (15 ng) was used for immunoprecipitation by the anti-HMGA1 antibody (lanes 3, 6, and 9, control). The faint band in lane 8 at 43 kDa represents non-specific signal. A representative of three separate assays is shown for each condition.

In an attempt to determine whether and/or how HMGA1 regulates *INS* gene expression, we first analyzed whether HMGA1 interacts with PDX-1 and MafA without DNA. *In vitro*-translated ^35^S-labeled proteins PDX-1 and MafA were analyzed for their ability to be specifically retained by a GST-HMGA1 affinity resin. As shown in Figure 2A, PDX-1 and MafA were both retained by GST-HMGA1, but not GST alone, indicating that both these factors physically interact with HMGA1 *in vitro*, in the absence of DNA. The high specificity of the interaction of HMGA1 with PDX-1 and MafA was highlighted by the observation that no protein–protein contact was detected between HMGA1 and Neuro D (Figure 2A), a transactivator that is critical for *INS* gene expression in both human and mouse beta cells (45). To verify whether this protein–protein interaction *in vitro* could occur also *in vivo*, we performed co-immunoprecipitation studies using an anti-HMGA1 antibody immobilized on protein A-Sepaharose beads. As shown in Figure 2B, immunoprecipitation of HMGA1 from INS-1 nuclear extracts specifically pulled down PDX-1 and MafA, as shown by Western Blot analysis with well-characterized anti-PDX-1 or anti-MafA antibodies. These findings unequivocally demonstrate that HMGA1 physically interacts with PDX-1 and MafA both *in vitro* and *in vivo*, and suggest that physical interactions between these nuclear factors may constitute a fundamental prerequisite for functional cooperation between HMGA1, PDX-1, and MafA.

### HMGA1 potentiates the activities of PDX-1 and MafA on *INS* gene transcription

**Figure 3:**
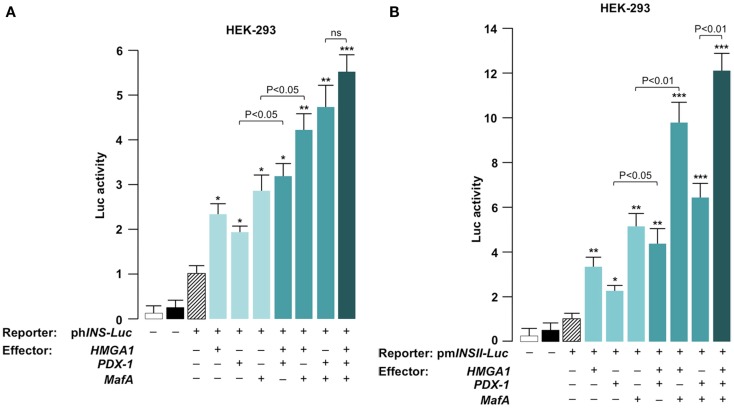
**Functional significance of HMGA1, PDX-1, and MafA for *INS* gene transcription**. HEK-293 cells were cotransfected with 1 μg of human (ph*INS-Luc*) **(A)** or mouse (pm*INSII-Luc*) **(B)** *Luc* reporter plasmids, in the absence or presence of effector vectors for HMGA1, PDX-1, and MafA (0.5 μg each), either alone or in double or triple combinations. Data represent the means ± SE for three separate experiments; values are expressed as factors by which *Luc* activity increased as compared with the level of *Luc* activity obtained in transfections with reporter vector alone (slashed bar), which is assigned an arbitrary value of 1. Open bar, mock (no DNA); black bar, pGL3-basic (vector without an insert). *p < 0.05; **p < 0.01; ***p < 0.001 versus control (slashed bar).

We next determined whether HMGA1, PDX-1, and MafA functionally cooperate to activate *INS* transcription. HEK-293 cells were cotransfected transiently with *Luc* reporter plasmids containing human or mouse *INS* gene promoter and various effector vectors. HEK-293 cells were ideally suited for studying the effects of these proteins on transcription since they express only low levels of HMGA1 and no PDX-1 or MafA. As shown in Figure 3A, forced expression of single transcription factors HMGA1, PDX-1, and MafA in HEK-293 cells activated the human *INS* promoter to levels of two to threefold above the background with the recombinant reporter vector (*phINS-Luc*) alone. An ulterior increase in promoter activity was found in the presence of a combination of two of these transcription factors, while a further additive effect was observed in the promoter activity of *phINS-Luc* in cells overexpressing all three proteins (HMGA1, PDX-1, and MafA) together. Comparable or better results were obtained in HEK-293 cells when the mouse *Ins* promoter (*pmInsII-Luc*) was utilized for transactivation assays (Figure 3B). Differences in the activity of human and mouse *INS* gene promoters in HEK-293 cells may reflect the differences between humans and mice in terms of transcriptional activation of genes associated with pancreatic function (38). The greater increase in promoter activity of the *pmInsII-Luc* compared to the *phINS-Luc* in cells overexpressing MafA is likely attributable to the greater number of MafA binding sites within the mouse *InsII* promoter. Given that full sequence analysis with MatINspector predicted no HMGA1 binding sites in the human and mouse *INS* promoters, these results indicate that HMGA1 acts in concert with PDX-1 and MafA to regulate the expression of human and mouse *INS* genes through a mechanism that does not require its direct binding to DNA. Similar findings, indicating that HMGA1 acts to regulate the expression of estrogen responsive genes through a mechanism that does not require direct binding to DNA, have been reported elsewhere (46).

**Figure 4:**
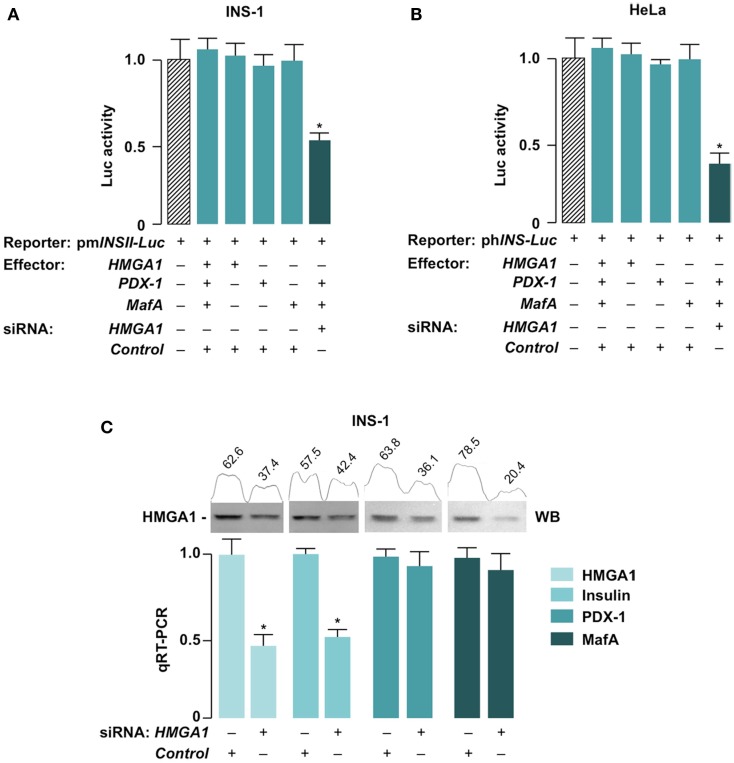
**Suppression of *INS* promoter and mRNA by siRNA to HMGA1**. INS-1 **(A)** and HeLa **(B)** cells were incubated without or with siRNA targeting HMGA1 (100 pmol) and transfected thereafter with 1 μg of mouse (pm*INSII-Luc*) or human (ph*INS-Luc*) *Luc* reporter plasmid, respectively, either in the absence or presence of effector vectors (0.5 μg each) for HMGA1, PDX-1, and MafA. Values are expressed relative to the *Luc* activity obtained in transfections with the *Luc* reporter plasmid alone (slashed bar) that is assigned an arbitrary value of 1. Results are the means ± SE of triplicates from three independent transfections. *p < 0.05 versus control (slashed bar). **(C)** INS-1 cells were transfected with siRNA against HMGA1 or a non-targeting control siRNA, and mRNA levels for HMGA1, Insulin, PDX-1, and MafA were measured by quantitative RT-PCR (qRT-PCR). Results are the mean ± SEM of at least three separate transfections, each in triplicate. *p < 0.05 versus the relative control. Western blots (WB) of HMGA1, either before or after HMGA1 siRNA treatment, are shown in the autoradiograms and are from four independent experiments. Densitometric slot blot analysis, using the ImageJ software program, is shown. Numbers on the peaks are the size of the corresponding slot as a percentage of the total size of the two slots in each condition.

We next tested whether HMGA1 is required for high transactivation activity of PDX-1 and MafA on *INS* gene in beta cells. For this purpose, we performed HMGA1 knockdown in INS-1 cells with siRNA approaches. As shown in Figure 4A, *INS-luc* activity failed to increase in INS-1 cells with overexpression of HMGA1, PDX-1, and MafA separately or in combination, probably because of the saturating levels of endogenous HMGA1, PDX-1, and MafA in this insulin-producing cell line. However, the promoter activity of *pmInsII-Luc* was significantly blunted in INS-1 cells with HMGA1 knockdown (Figure 4A), indicating that HMGA1 is required for full activation of *INS* transcription *in vivo*. A similar reduction in *INS* promoter activity was observed in HeLa cells that naturally express HMGA1 but not PDX-1 and MafA. In this case, whereas ectopic expression of PDX-1 and MafA, together, only weakly activated the expression of the reporter vector *phINS-Luc*, knockdown of endogenous HMGA1 significantly prevented this activation (Figure 4B). Consistently with the results reported above, and in agreement with our previous findings that pancreatic insulin mRNA transcripts were reduced in *HMGA1*-knockout mice (38), *INS* mRNA levels were significantly lower in INS-1 cells in the presence of siRNA directed against HMGA1, whereas mRNA abundances of PDX-1 and MafA were not altered by siRNA-induced down-regulation of HMGA1 (Figure 4C).

Taken together, these data confirm and extend previous observations and provide strong evidence that HMGA1 is required for full transactivation of the *INS* gene in beta cells.

### Effects of HMGA1 on glucose-induced *INS* gene expression and insulin secretion

**Figure 5:**
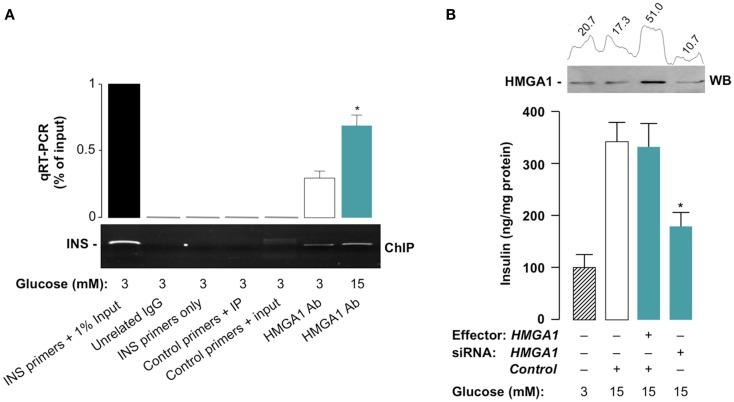
**Functional significance of HMGA1 for glucose-induced *INS* gene expression and insulin secretion**. **(A)** ChIP of the rat *Ins* promoter gene in INS-1 cells treated with either basal (3 mM) or stimulatory (15 mM) glucose concentrations, using an anti-HMGA1 specific antibody (Ab). A representative assay is shown, together with qRT-PCR of ChIP-ed samples. *p* < 0.05 versus control (white bar). **(B)** Insulin secretion from HMGA1 siRNA-treated INS-1 cells. Results are the mean ± SEM for three independent experiments conducted in triplicate. *p* < 0.05 versus control (white bar). A representative WB of HMGA1 is shown in the autoradiogram, together with densitometric slot blot analysis in each condition.

High glucose levels not only trigger a rapid secretion of insulin stored in the beta-cell but also stimulate the synthesis of new insulin molecules by inducing *INS* gene transcription. As reported previously, one of the mechanisms whereby glucose stimulates *INS* gene transcription in pancreatic beta cells is via activation and nuclear translocation of the transcription factor PDX-1 (25, 47), in addition to the up-regulation of MafA transcription (48). In the light of the data presented above, indicating the cooperative interactions between HMGA1, PDX-1, and MafA, we investigated whether HMGA1 plays a role in glucose-induced insulin transcription. To this end, we first carried out ChIP experiments in INS-1 cells at basal (3 mM) and stimulatory (15 mM) glucose concentrations. Results showed that binding of HMGA1 to the A3/A4 region of the *INS* gene promoter was low in INS-1 cells incubated in low glucose condition, while this binding was significantly increased under high glucose conditions (Figure 5A). These findings demonstrate that binding of HMGA1 to the *INS* promoter is stimulated by high glucose and indicate that HMGA1 could mediate the *INS* transactivation ability of high glucose. To further support this conclusion, HMGA1 siRNA-treated INS-1 cells were used to test the effects of glucose on insulin release into the culture medium. As shown in Figure 5B, a significant increase in insulin secretion was obtained when the medium glucose content was increased from 3 to 15 mM. No further increase in insulin secretion was observed by transfection with HMGA1 expression vector, most probably due to the endogenous HMGA1 present in INS-1 cells. However, pretreatment of INS-1 cells with HMGA1 siRNA resulted in a significant reduction in glucose-induced insulin secretion into the incubation medium (Figure 5B), confirming the central importance of HMGA1 in this scenario. Taken together, these results provide new insights into the molecular mechanisms regulating *INS* gene expression and emphasize the role of HMGA1 as an essential molecule necessary for proper beta-cell insulin secretion.

## Discussion

Functional defects in the pancreatic beta-cell result in type 2 diabetes mellitus, a metabolic disorder in which, for various reasons, beta-cell insulin secretion may be profoundly affected at the multiple stages of the natural history of the disease. At the initial stage, peripheral insulin resistance increases demands of insulin secretion to stabilize blood sugar, which requires increased insulin transcription and translation (49). Yet sustained high glucose and insulin secretion eventually induce stress responses in beta cells to attenuate insulin production and secretion. Supporting the complexity and the importance of insulin production and secretion, genome-wide association studies have shown that most of the multiple susceptibility genes that facilitate diabetes development are involved in controlling beta-cell mass and function (1, 2). Thus, understanding factors that regulate insulin transcription and translation is critical to prevent or delay the development of type 2 diabetes.

By binding to AT-rich regions in the minor groove of DNA, HMGA1 contributes importantly to the transcriptional activation of many mammalian genes. By itself, HMGA1 has no intrinsic transcriptional activity; rather, it binds DNA to modulate its conformation so that other transcription factors can be recruited and assembled at the transcription initiation site to regulate transcription (34–37). These findings suggest that HMGA1 acts as a general nuclear factor to regulate gene expression. Indeed, promoters of genes that are expressed in a tissue-specific fashion are often regulated by the combination of tissue-specific and ubiquitous nuclear proteins, where the ubiquitous factor can support and facilitate the action of tissue-specific transcription factors (36, 50). As a part of an investigation into the genetic basis of insulin resistance syndromes and diabetes, we previously reported that loss of HMGA1 expression considerably decreased beta-cell insulin expression and severely reduced insulin secretion, causing a phenotype with hypoinsulinemia and glucose intolerance (38). Interestingly, pancreatic islets from *Hmga1*-knockout mice were nearly 80% smaller compared with wild-type islets, indicating that decreased insulin secretion in this mutant mouse model was dependent, at least in part, on reduced beta-cell mass, and suggesting that HMGA1 may play a direct role in pancreatic islet development and insulin production.

In this study, we provide direct evidence that HMGA1 is required for proper transcription of the *INS* gene in culture cells. We show that functional integrity of HMGA1 was necessary for full transactivation of the *INS* promoter by PDX-1 and MafA in cells that do not normally produce insulin. In contrast, repression of endogenous HMGA1 protein function adversely affected both PDX-1- and MafA-induced transactivation of the *INS* gene in insulin-producing cells. These findings demonstrate that HMGA1 plays significant roles in the transcriptional activities of these nuclear factors in the context of the *INS* gene. Underscoring the importance of this biochemical activity, our ChIP-qRT-PCR-based analysis showed that glucose upregulates HMGA1’s activity to the endogenous *INS* chromosomal locus. It is likely also that after a glucose challenge a deficit in HMGA1 may dampen *INS* gene transcriptional rate, thereby reducing insulin production.

Functional cooperation between HMGA1, PDX-1, and MafA in the transactivation of the *INS* promoter could be mediated by HMGA1-induced changes in DNA structure, thereby triggering a chain of molecular events that enhances the affinity of PDX-1 and MafA for their target DNA sequence and perhaps for other DNA-binding proteins that bind in the immediate vicinity, thus promoting the formation of an active transcription complex to which coactivators are recruited. While the biological relevance of these findings with regard to type 1 and type 2 diabetes remains to be clarified, it is tempting to hypothesize that a putative defect in HMGA1, by adversely affecting binding of PDX-1 and MafA to the *INS* gene, under either basal or glucose-stimulated conditions, may impair *INS* gene transcription, leading to decreased insulin synthesis and secretion. Consistent with the high sequence homology of the human and mouse *INS* genes (7), our data on *in vitro INS* gene transactivation demonstrate a striking similarity between the two species. This is particularly noteworthy in the light of several observations in the literature, indicating that substantial differences exist in pancreatic islet morphogenesis and function between humans and mice (38, 51, 52). Thus, caution is required when generalizing these results, as well as additional confirmation in future studies.

In conclusion, our data in the present work consistently suggest that HMGA1 may act as a glucose sensor that facilitates activation of the *INS* gene and insulin release. In addition, they further support the notion that HMGA1 is an architectural element tightly linked to glucose metabolism and metabolic disorders (38, 44, 53–60). Future studies on the interplays among HMGA1, PDX-1, and MafA might be useful in understanding the molecular basis of clinical phenotypes in certain clinical conditions where insulin secretion becomes compromised (i.e., diabetes mellitus and other categories of glucose intolerance). Understanding these mechanisms should augment our capacity to identify novel therapeutic targets for the prevention and treatment of these diseases.

## Author Contributions

Performed and designed the experiments (Biagio Arcidiacono, Stefania Iiritano); drafting of the manuscript (Eusebio Chiefari, Daniela Patrizia Foti, Antonio Brunetti); critical revision and editing of the manuscript (Antonio Brunetti, Guoqiang Gu, Francesco S. Brunetti). All authors have approved the submitted version.

## Conflict of Interest Statement

The authors declare that the research was conducted in the absence of any commercial or financial relationships that could be construed as a potential conflict of interest.
